# Disposal of the Dead

**Published:** 1896-11

**Authors:** 


### EDITORIAL.

The office of the Business Manager of THE JOURNAL is Nos. 311 and 312 Fitten Building.

The editors are not responsible for views expressed by contributors.

Articles for publication should reach this office not later than the 15th inst.

Address all communications, and make all remittances payable to THE ATLANTA MEDICAL AND SURGICAL JOURNAL, Atlanta, Ga.

Reprints of articles will be furnished, when desired, at a nominal cost. Requests for the same should always be made *on the manuscript*.

IF A SUBSCRIBER NOTIFIES US TO STOP HIS JOURNAL AND IT IS NOT DONE, OR, IF HE ORDERS HIS ADDRESS CHANGED AND IT IS NOT, HE MAY KNOW THAT WE DID NOT GET HIS ORDER. MOVING TO ANOTHER ADDRESS WITHOUT NOTIFYING US, DOES NOT EXCUSE HIM FROM PAYING HIS BILL.

### DISPOSAL OF THE DEAD.

At present it may be said, generally, that in civilized countries there are three methods of burial, each having a different end in view, or at least having different results as regards the changes of the body after death. These are the closing up of the body in more or less hermetically sealed coffins, composed of very indestructible material, placed in vaults, or deeply in the earth; the enclosing of the body in coffins of light material, so constructed that the effect of burial is to cause the walls to fall in and permit the access of earth to its interior—these also are deposited deeply; and cremation.

Once the mind has become accustomed to the idea, little objection can be taken to the last, beyond the possibility of difficulties owing to the destruction of evidence in connection with the cause of death. But against the first, which seems to be that almost universally in vogue in this locality, several very strong arguments may be urged. It is nasty in the extreme, so much so that the mind refuses, except under compulsion, to consider the condition of the mortal remains of once dear friends which are passing through the unnecessarily long-continued and unnecessarily revolting changes of reconversion to their pristine elements. Such is not "earth to earth" burial; its *raison d'être* is separation of earth from earth. As corollaries of this are the fact that graveyards cover an unnecessary proportion of available land, and that the soil in their vicinity is unnecessarily objectionable for residential purposes.

The second method is that which will in the future, when doctors and others have properly educated the community, probably take its place as the least objectionable of the three. It is the true "earth to earth" burial, for the idea upon which it is founded is the speediest possible contact of earth and body, compatible with the decencies of burial. But even it, as now employed, is not perfect in detail, for while the coffin is made collapsible and perishable, the body is still wrapped in the linen of ordinary wear. A special cloth of short fibre, composed of pith or some other rapidly disintegrating substance, might no doubt be produced for this purpose.

We know now that the bacteria of the soil play a chief part in its chemistry, and that they are most active near the surface. Sir Seymour Haden has found by experiments carried on during the last ten years that, buried at the minimum depth permitted by the English government, 4½ feet, three or four years were required for the complete resolution of uncovered bodies; at the depth of one foot a year and a half was sufficient for the larger, and one year or less for the smaller animals. A body simply laid on the ground, with a covering of earth a foot thick, became, except the bones, completely resolved into oxygen, hydrogen, carbon and other innocuous products necessary for the food of plants in a yet shorter period.

Dr. Poore, of London, contends at the same time that, with proper shallow burial, there is no reason to fear an excessive demand for graveyards, for when properly planted the same land may, with safety, be used again at intervals of about ten years. Thus an acre would suffice forever for a population of 10,000 persons.

